# Novel Features of the Prenatal Horn Bud Development in Cattle (*Bos taurus*)

**DOI:** 10.1371/journal.pone.0127691

**Published:** 2015-05-20

**Authors:** Dominique Judith Wiener, Natalie Wiedemar, Monika Maria Welle, Cord Drögemüller

**Affiliations:** 1 Institute of Animal Pathology, Vetsuisse Faculty, University of Bern, Bern, Switzerland; 2 DermFocus, Vetsuisse Faculty, University of Bern, Bern, Switzerland; 3 Institute of Genetics, Vetsuisse Faculty, University of Bern, Bern, Switzerland; INRA, FRANCE

## Abstract

Whereas the genetic background of horn growth in cattle has been studied extensively, little is known about the morphological changes in the developing fetal horn bud. In this study we histologically analyzed the development of horn buds of bovine fetuses between ~70 and ~268 days of pregnancy and compared them with biopsies taken from the frontal skin of the same fetuses. In addition we compared the samples from the wild type (horned) fetuses with samples taken from the horn bud region of age-matched genetically hornless (polled) fetuses. In summary, the horn bud with multiple layers of vacuolated keratinocytes is histologically visible early in fetal life already at around day 70 of gestation and can be easily differentiated from the much thinner epidermis of the frontal skin. However, at the gestation day (gd) 212 the epidermis above the horn bud shows a similar morphology to the epidermis of the frontal skin and the outstanding layers of vacuolated keratinocytes have disappeared. Immature hair follicles are seen in the frontal skin at gd 115 whereas hair follicles below the horn bud are not present until gd 155. Interestingly, thick nerve bundles appear in the dermis below the horn bud at gd 115. These nerve fibers grow in size over time and are prominent shortly before birth. Prominent nerve bundles are not present in the frontal skin of wild type or in polled fetuses at any time, indicating that the horn bud is a very sensitive area. The samples from the horn bud region from polled fetuses are histologically equivalent to samples taken from the frontal skin in horned species. This is the first study that presents unique histological data on bovine prenatal horn bud differentiation at different developmental stages which creates knowledge for a better understanding of recent molecular findings.

## Introduction

It is not by chance that the family of *bovidae* that includes bison, African buffalo, water buffalo, antelopes, gazelles, sheep, goats, muskoxen, and domestic cattle is also called “horn-bearer” in German language, as one of the typical characteristics of *bovidae* is the presence of a pair of horns. In contrast to antlers in *cervidae* [1], horns in *bovidae* are permanent and consist of an outer permanent sheath of keratin and a bony pneumatized core [2].

In domesticated ruminants like cattle there is evidence for the existence of hornless (polled) animals until back to ancient times, as for example shown in several Old Egyptian tomb sceneries [3]. In modern cattle production, the replacement of traditional tie stalls with free stalls during the last decades has led to an increasing rate of dehorning. Due to animal welfare discussion in context with dehorning [4,5] selected breeding of polled cattle has become more and more popular. The underlying genetic cause of this autosomal dominant Mendelian trait has been studied by several groups [6–14].

There are two known non-coding, highly likely regulatory mutations leading to the absence of horn growth, both are located in 280 kb region on cattle chromosome 1 [15–18]. In beef and dual purpose breeds a 212 bp insertion-deletion causes absence of horns [15–17] and in the Holstein dairy cattle an 80 kb duplication is perfectly associated with polledness [15–18]. In some breeds both mutations are prevalent and segregate independently [17]. Additionally, sporadic *de novo* mutations affecting horn growth in the absence of the known polled mutations are described [19,20]. Preliminary data suggest that the expression of a long noncoding RNA is altered by the two known genomic mutations causing polledness [16,17]. Therefore it was implicated that this uncharacterized specific transcript, which is known in cattle and buffalo only, represents an important prerequisite for horn bud formation.

Finally, the precise functional consequences of the polled mutations are still not completely understood. Gene expression studies in fetal biopsies of frontal skin and horn buds indicate the involvement of different genes in horn bud development. Actually, it is not known which genes are primarily causative for bovine horn bud formation and which expression changes are secondary consequences of this event [16,17]. In domestic goats, it was demonstrated that a genomic deletion of 11.7 kb causing polledness affects the transcription of at least two adjacent genes including a non-coding RNA gene which is not related to the one identified in cattle [21].

Whereas genetic background of horn growth in cattle has been studied in depth, little is known about the morphological features of bovine horn growth. Using tissue transplantation it was shown, as reviewed by Capitan et al. [19], that (i) the bony core is not an outgrowth of the skull but originates from a separated center of ossification located in the dermis and hypodermis of the calves horn bud; (ii) the keratinization of the horn bud epidermis does not induce ossification of the underlying dermis and hypodermis and conversely, thus both phenomena are probably programmed during embryogenesis; (iii) the ossifying hypodermal tissue induces the frontal bone to grow upward and to form the base of the horn spike, then it fuses with the skull by dissolving it locally [22]. Thus, it was concluded that horn development is the result of differentiation and remodeling of various tissues originating from two distinct germ layers: ectoderm and mesoderm [22]. Later on, histological changes have been described in fetuses with a neck-rump length of 5.2 cm, which show a thickening of the epidermis in the region of the horn bud [23]. During development the horn bud region gets indented macroscopically while histologically hair follicles, sweat glands and sebaceous glands are visible. In fetuses with at neck-rump length around 61 cm the further thickening of the epidermis is accompanied by an atrophy of the adnexal structures. Nevertheless macroscopically a whorl of hairs is visible at the time of birth [23].

Capitan et al. [20] studied the “polled and multisystemic syndrome”, a developmental disorder which is caused by a 3.7 Mb deletion on chromosome 2 and which is characterized by polledness, facial dysmorphism, growth delay, chronic diarrhea, premature ovarian failure and variable neurological and cardiac anomalies. These authors compared horn buds and frontal skin from horned (wild type) fetuses with tissue from the region of the horn bud and frontal skin from fetuses affected by the “polled and multisystemic syndrome” at gestation day (gd) 90. They report multiple layers of vacuolated keratinocytes and clusters of dermal cells displaying glandular/ductal differentiation in the wild type horn bud, which are absent in the neighboring frontal skin. In addition, skin gland primordia, which are not yet detectable in normal frontal skin, are visible in the region of the horn bud at this stage [20]. Allais-Bonnet et al. [16] describes one additional feature in wild type horn buds at gd 90: the absence of hair follicle germs, which at this stage are already present in frontal skin. In genetically polled fetuses the tissue in the region of the horn bud was shown to be identical with regular frontal skin at gd 90 [16].

The aim of this study was to analyze the morphological development of prenatal horn buds using bovine fetuses between ~70 and ~268 days of gestation. In addition horn buds of genotypic wild type and mutant polled fetuses of the same developmental stages were compared. To the best of our knowledge, this is the first study that investigated the histological changes in fetal horn buds in different developmental stages and with our results we gained new knowledge about the developing fetal horn bud.

## Results

### Histological appearance

#### Gestation day 70 and 83 (2–3 months of gestation)

**Fig 1:**
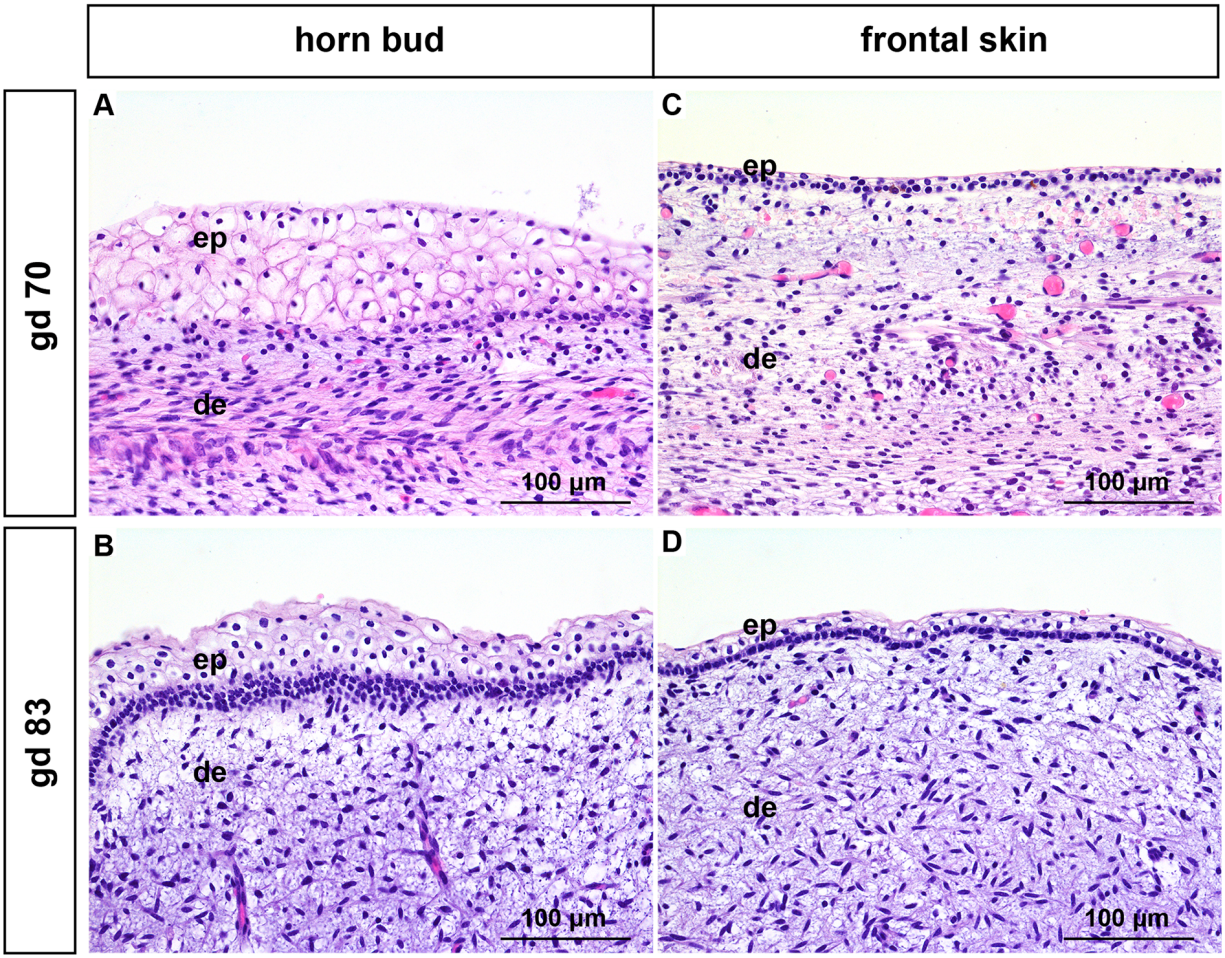
Histological analysis of horn buds and frontal skin from wild type fetuses (gd 70 and 83). (A) and (B): Horn buds with multiple layers of vacuolated keratinocytes. (C) and (D): Frontal skin. Haematoxylin and eosin. Gd = gestation days, ep = epidermis, de = dermis.

The epidermis in the region of the horn bud is up to 7 layers thick and the keratinocytes are vacuolated (Fig 1A and 1B). In contrast, the epidermis of the frontal skin is thinner with up to 3 layers of vacuolated keratinocytes (Fig 1C and 1D). The dermis in both the horn bud and the frontal skin is composed of immature collagen. No adnexal structures are present.

#### Gestation day 115 and 140 (3–4 months of gestation)

**Fig 2:**
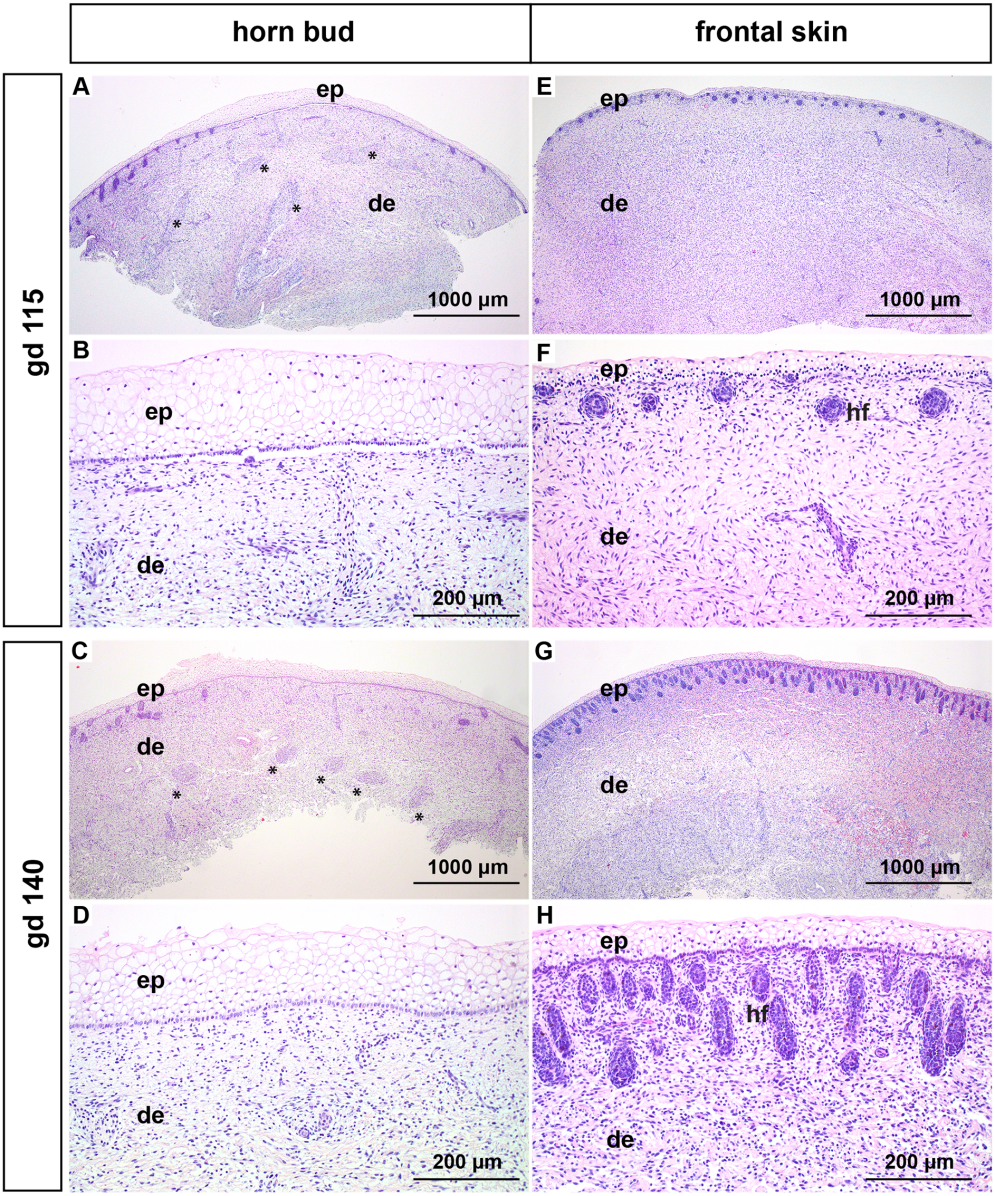
Histological analysis of horn buds and frontal skin from wild type fetuses (gd 115 and 140). (A-D): Horn buds with multiple layers of vacuolated keratinocytes. Note the absence of hair follicles below the horn bud in (A-D). (A) and (C): Note thick nerve bundles in the dermis below the horn bud (black stars). (B) and (D): Represent magnifications (A) and (C). (E-H): Frontal skin. (E) and (G): Note the absence of thick nerve bundles in the dermis. (F) and (H) represent magnifications of (E) and (G). Haematoxylin and eosin. Gd = gestation days, ep = epidermis, de = dermis, hf = hair follicles.

The epidermis in the region of the horn bud is up to 12 layers thick and the keratinocytes are vacuolated (Fig 2A–2D). The epidermis of the frontal skin shows up to 4 layers of vacuolated keratinocytes (Fig 2E–2H). Below the horn bud no hair follicles are present, however, thick nerve bundles are visible in the dermis (Fig 2A and 2C). Thick nerve bundles are not present in the dermis of the frontal skin (Fig 2E and 2G). Immature hair follicles are present in the superficial dermis at the border of the horn buds as well as in the superficial dermis from samples derived from the frontal skin. The immature hair follicles are present in the hair germ (Fig 2F) and hair peg stage (Fig 2H), respectively.

#### Gestation day 155 and 172 (5–6 months of gestation)

**Fig 3:**
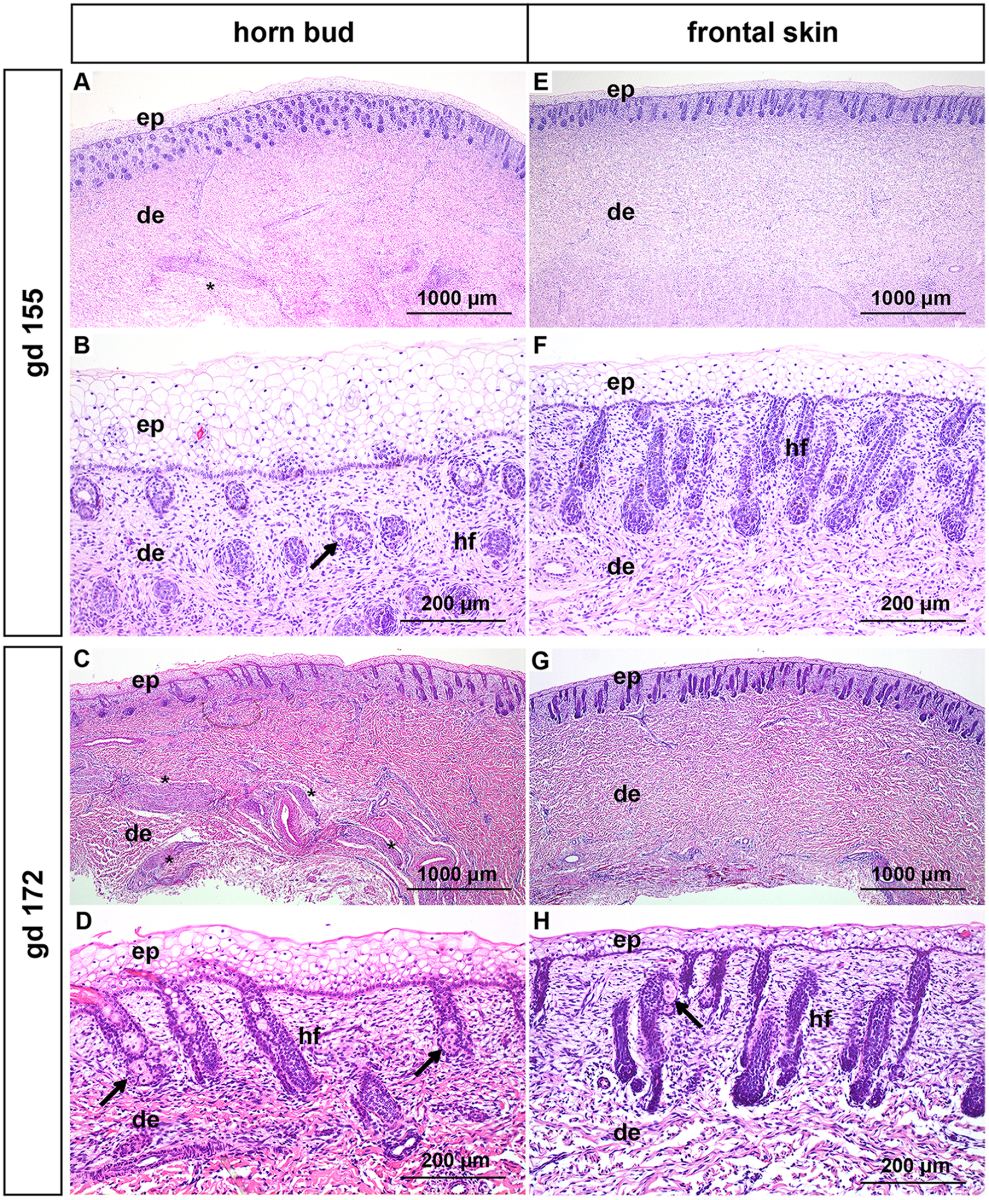
Histological analysis of horn buds and frontal skin from wild type fetuses (gd 155 and172). (A-D): Horn buds with multiple layers of vacuolated keratinocytes. (A) and (C): Note thick nerve bundles in the dermis below the horn bud (black stars). (B) and (D): Represent magnifications of (A) and (C). Note presence of sebaceous glands (black arrows). (E-H): Frontal skin. (E) and (G): Note the absence of thick nerve bundles in the dermis. (F) and (H): Represent magnifications of (E) and (G). (H): Note the presence of sebaceous glands (black arrows). Haematoxylin and eosin. Gd = gestation days, ep = epidermis, de = dermis, hf = hair follicles.

The epidermis in the region of the horn bud is up to 12 layers thick and the keratinocytes are vacuolated (Fig 3A–3D). The epidermis of the frontal skin shows up to 6 layers of vacuolated keratinocytes (Fig 3E–3H). Hair follicles are present in the dermis of all samples (below the horn bud and in the frontal skin) in the bulbous peg stage (Fig 3A–3H). Sebaceous glands are present at gd 155 as well as at gd 172 in the area of the horn bud and at gd 172 in the area of the frontal skin (Fig 3B, 3D and 3H, black arrows). Thick nerve bundles are present in the dermis in the area of the horn bud (Fig 3A and 3C) and are absent in the frontal skin (Fig 3E and 3G). The nerve bundles are more prominent compared to earlier developmental stages.

#### Gestation day 212, 230 and 268 (7–8 months of gestation)

**Fig 4:**
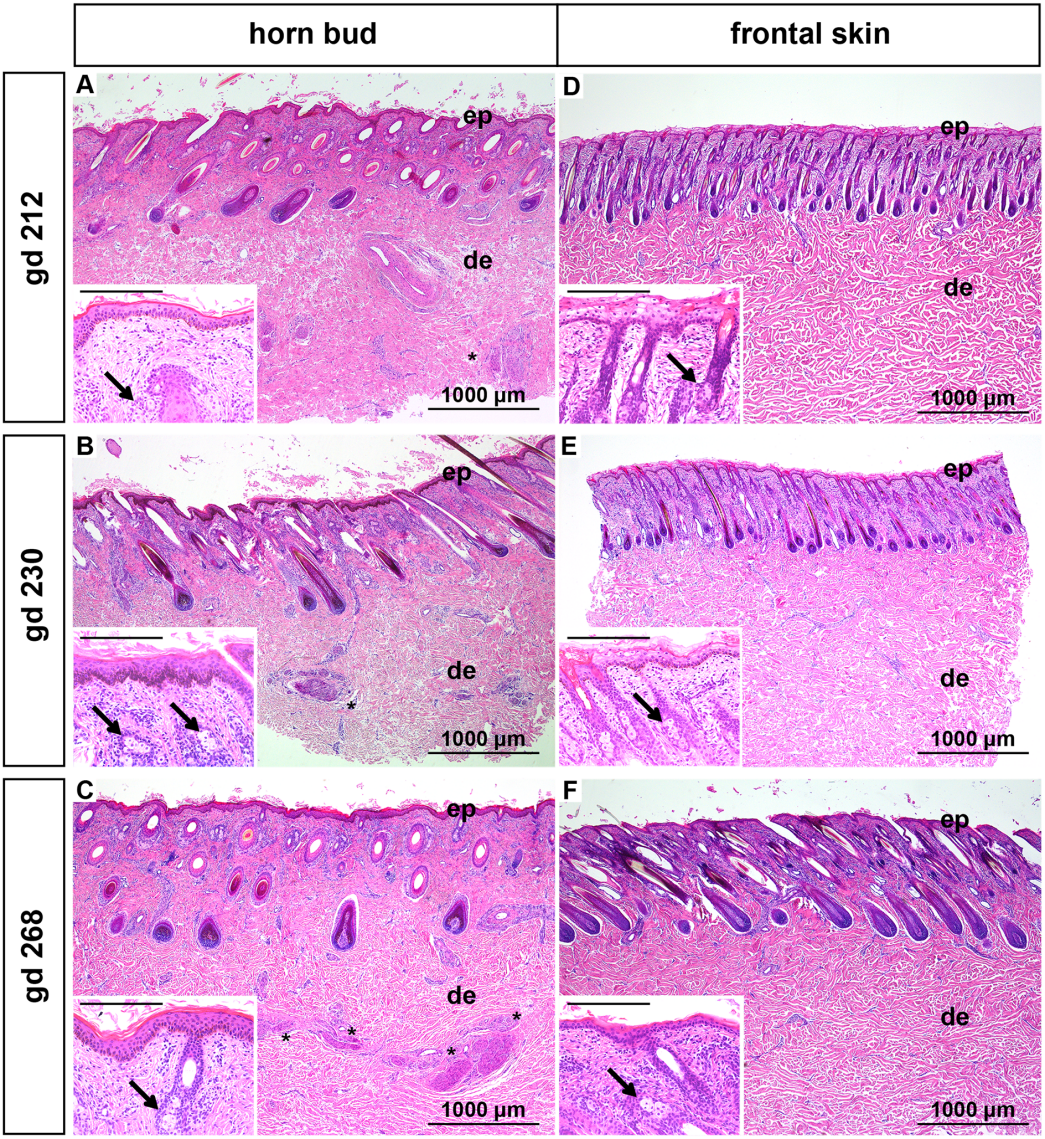
Histological analysis of horn buds and frontal skin from wild type fetuses (gd 212, 230 and 268). (A-C): Horn buds. Note differentiated epidermis and thick nerve bundles in the dermis below the horn bud (black stars). Insets show well-differentiated keratinocytes in the epidermis and sebaceous glands (black arrows). (D-F): Frontal skin. Note the absence of thick nerve bundles in the dermis. Insets show immature keratinocytes in (D) and (E) and well-differentiated keratinocytes in (F). Note sebaceous glands in the insets (black arrows). Haematoxylin and eosin. Insets: Scale bar represents 200μm. Gd = gestation days, ep = epidermis, de = dermis.

The number of layers of keratinocytes in the region of the horn bud is the same than in the frontal skin (Fig 4A–4C). The epidermis is well differentiated and keratinocytes are not vacuolated anymore (insets in Fig 4A–4C). In contrast, the keratinocytes of the epidermis derived from the frontal skin show vacuolization up to gd 230 (insets in Fig 4D and 4E). At gd 268 the epidermis of the frontal skin is well differentiated (inset in Fig 4F). Differentiated hair follicles as well as sebaceous glands and sweat glands are present in all samples (insets in Fig 4A–4H). Thick nerve bundles are visible in the dermis below the horn bud (Fig 4A–4C) and are absent in the frontal skin (Fig 4D–4F).

#### Mesenchymal structures in the dermis below the horn bud

**Fig 5:**
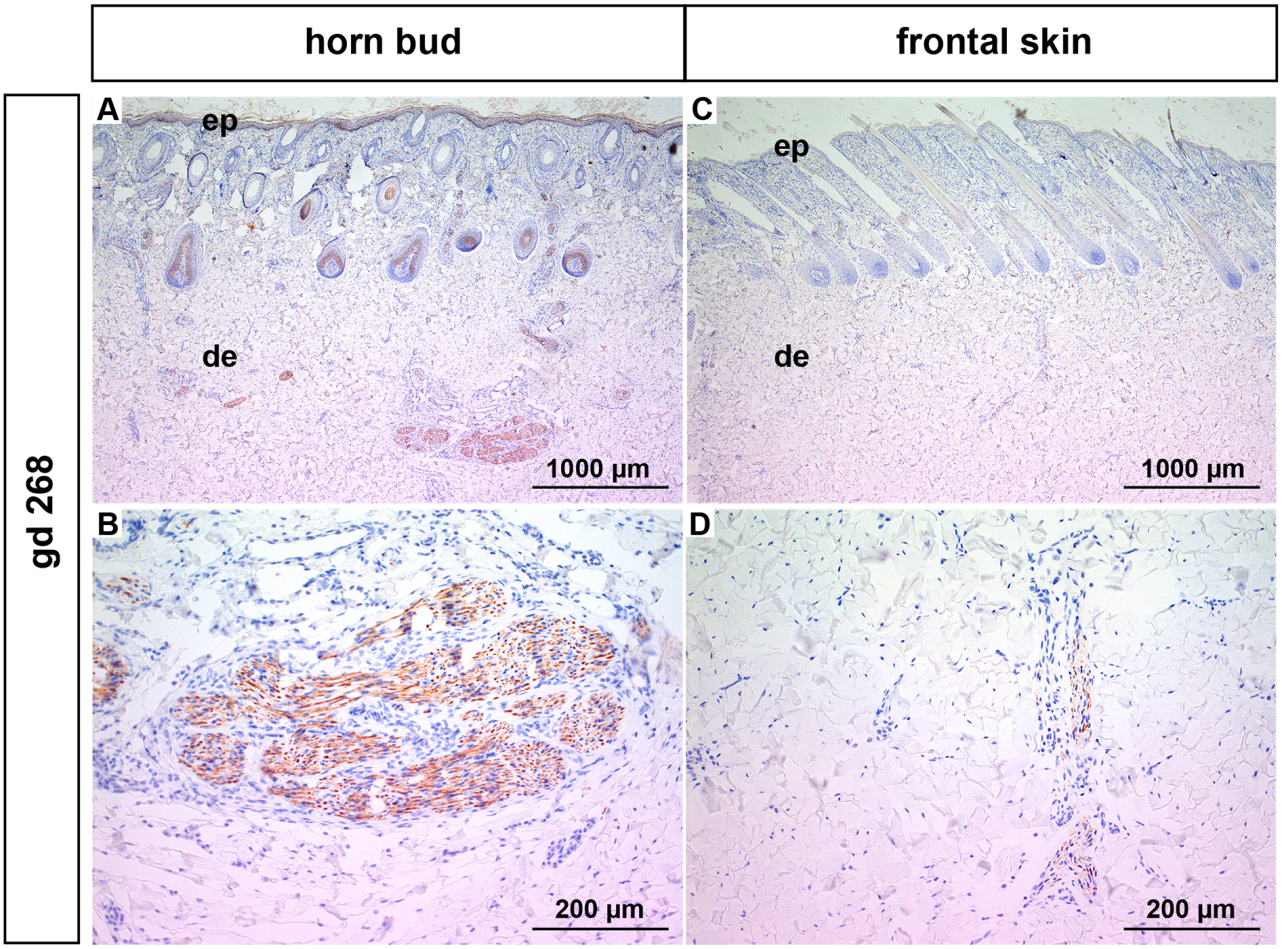
Neuron specific enolase staining from a wild type fetus (gd 268). (A) and (B): Horn bud. (B): Magnification of (A). Note positive staining of the thick nerve bundles in the dermis. (C) and (D): Frontal skin. (D): Magnification of (C). Note positive staining of nerve fibres of normal size in the dermis. Gd = gestation days, ep = epidermis, de = dermis.

**Fig 6:**
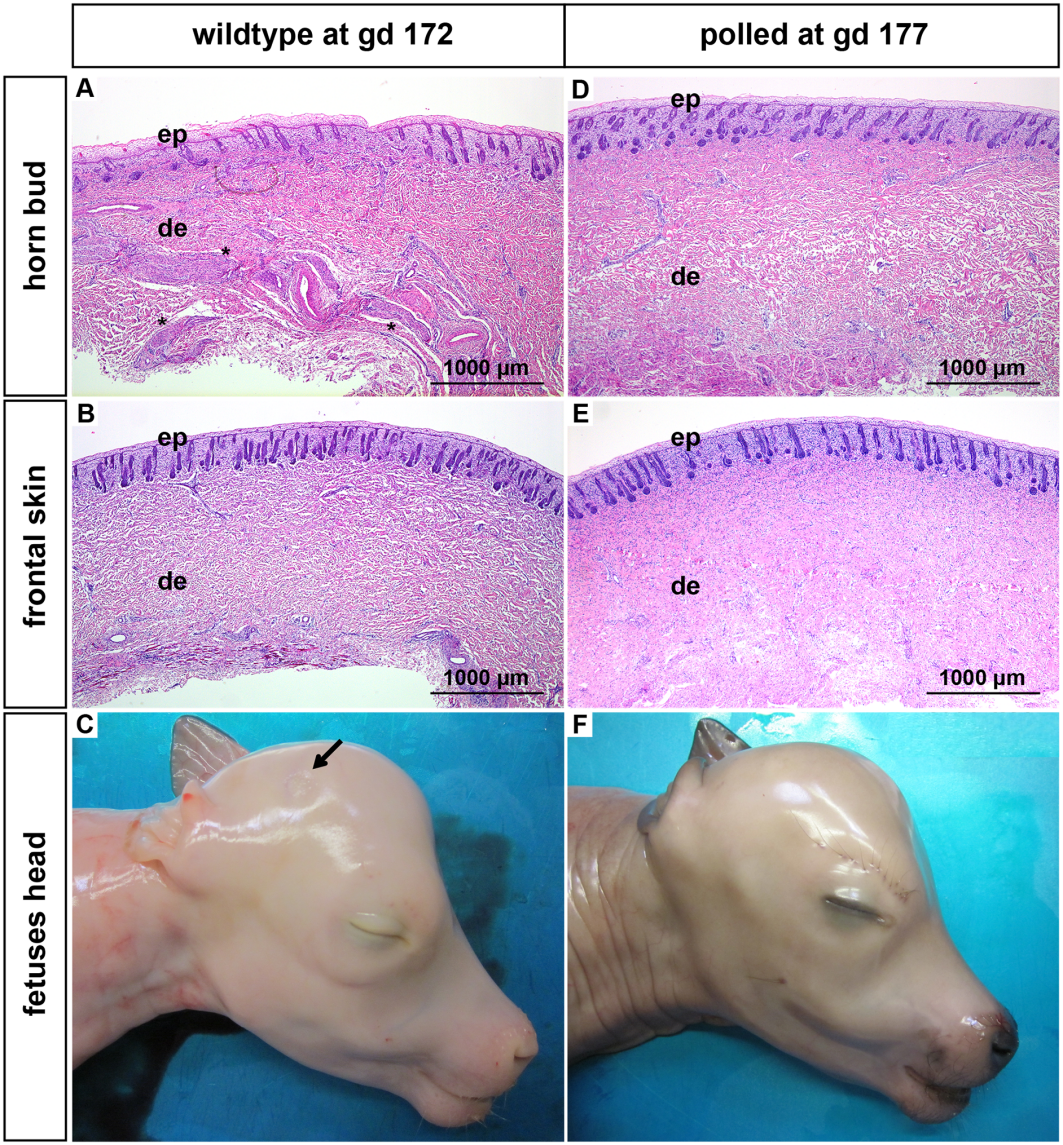
Features of horn buds and frontal skin from wild type and polled fetuses (gd 172 and 177). (A): Horn bud from a wild type fetus with multiple layers of vacuolated keratinocytes. Note presence of thick nerve bundles in the dermis below the horn bud (black stars). (B): Frontal skin from a wild type fetus. Note absence of thick nerve bundles in the dermis. (C): Macroscopic picture of a horn bud from a wild type fetus. Note indentation of skin (black arrow). Region of the horn bud (D) and frontal skin (E) from a polled fetus. Note absence of thick nerve bundles in the dermis. (F): Macroscopic picture of a polled fetus without indentation of the skin. Haematoxylin and eosin. Gd = gestation days, ep = epidermis, de = dermis.

Below the horn bud thick bundles of mesenchymal fibres appear early in gestation (gd 115) in the middle to deep dermis. These mesenchymal structures grow in size in the course of time and are prominent shortly before birth (Figs 2A, 2C, 3A, 3C and 4A–4C). Immunohistochemical stainings were performed with samples derived from fetuses at gd 140 (data not shown) and gd 268 (Fig 5), respectively. The mesenchymal structures stained positive for neuron specific enolase (NSE) (Fig 5A and 5B), identifying them as nerve fibers. Additional stainings for cytokeratin (CK), desmin and smooth muscle actin (SMA) were negative (data not shown). The thick nerve bundles were exclusively seen below the horn bud of the wild type and were not present in the frontal skin of the same fetuses (Figs 2E, 2G, 3E, 3G and 4D–4F). However, nerve fibers of normal size were seen in the dermis of the frontal skin (Fig 5C and 5D) as well as in the dermis below the horn bud in all developmental stages. To exclude that the presence of these thick nerve bundles is localization-dependent, horn buds from wild type fetuses were compared with samples derived from the same anatomic localization in age-matched, genetically hornless (polled) fetuses. The thick nerve bundles were present in the dermis below the horn bud of the wild type fetuses (Fig 6A) but were absent in the dermis of the frontal skin from the same fetus (Fig 6B) as well as in the dermis below the horn bud and the frontal skin in the polled fetuses (Fig 6D and 6E, respectively). In contrast to the wild type fetuses (horn bud is apparent as slightly indented area, Fig 6C), the region of the horn bud in polled fetuses, macroscopically and histologically, consists of normal skin and therefore cannot be distinguished from the surrounding area at all time-points (Fig 6F).

### Gross appearance

**Fig 7:**
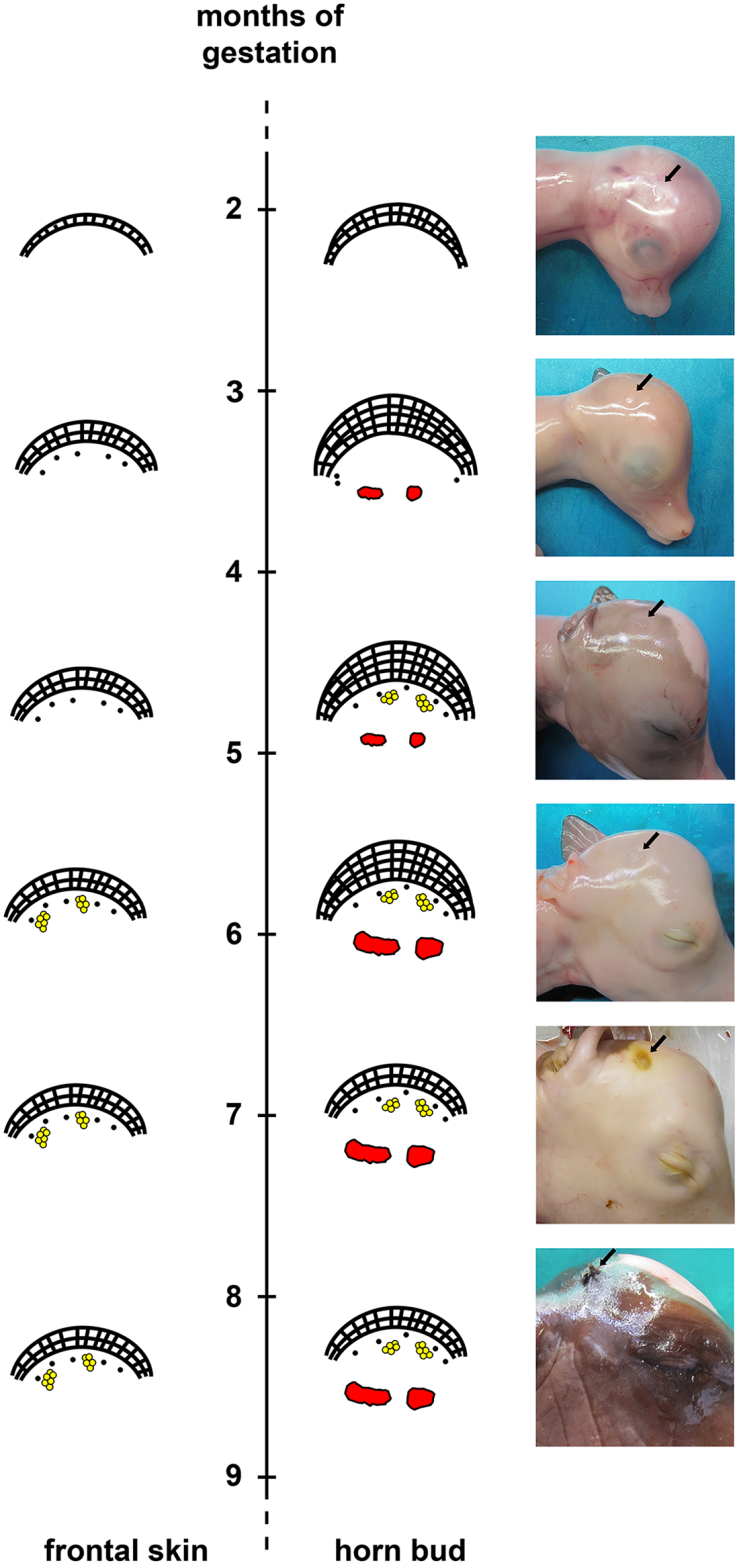
Schematic illustration of developmental stages of the horn bud and frontal skin in wild type fetuses. Horn bud: Multiple layers of keratinocytes are present between 2 and 6 months of gestation and hair follicles are lacking below the horn bud between 2 and 4 months. Note the appearance of thick nerve bundles below the horn bud at 3 months of gestation. Hair follicles and sebaceous glands are present below the horn bud at 4–5 months. Frontal skin: Note thin epidermis at all time-points. Sebaceous glands are present at about 5–6 months of gestation. Note absence of thick nerve bundles in the dermis. Macroscopic pictures: The black arrow depicts the region of the horn bud. Note presence of hair follicles at 6–7 months of gestation. Red = nerve fibres, yellow = sebaceous glands.

Macroscopically, at early development stages (2–3 months if gestation), the horn bud is barely visible and appears as a small, yellowish spot, which gets slightly indented at 3–4 months of gestation. Later, the horn bud is well visible as an indented area, but no hair follicles are macroscopically visible (5–6 months of gestation). At late-term gestation (7–8 months of gestation) the horn bud is well defined with densely packed hair follicles that form thick whorls of hairs (Fig 7).

## Discussion

We performed a histological description of the developing bovine horn bud at nine different time-points and compared the visible changes with normal frontal skin of the same fetuses. In comparison to previous studies which reported only histological sections at gd 90 [16,20] we present a detailed phenotypic description of the morphological changes from the suspected time-point of horn bud formation (gd 70) to the near end of the third trimester (gd 268).

In this study we compared different stages of development of the horn bud in wild type fetuses with frontal skin of the same fetus. In addition, we examined samples from the same regions of polled fetuses in similar developmental stages and compared them with the samples derived from the wild type fetuses. In the area of the horn bud as well as in the normal skin of fetuses at gd 70–172 vacuolated keratinocytes are visible (representing immature keratinocytes in the early stage of development), in contrast to mature, well differentiated keratinocytes in the later stage of fetal development (gd 212–268 and gd 268 in the horn bud area and the normal skin, respectively). Similar to previous reports [16,20] we noticed that horn buds show several layers of vacuolated keratinocytes (Figs 1A, 1B, 2A–2D and 3A–3D). However, these multiple layers of vacuolated keratinocytes at the site of the horn bud are visible only in the first five to six months of gestation. In later developmental stages the thickness of the epidermis in the horn bud area is comparable to the epidermis of the frontal skin (Fig 4A–4C). The previously described absence of hair follicle germs in the area of the horn bud [16,20] is apparent only from gd 115 to gd 140 (Fig 2A–2D). In later developmental stages, hair follicles are present below the horn bud as well as in the frontal skin (Figs 3A–3D and 4A–4D). Intriguingly, in the area of the horn bud sebaceous glands are present earlier (at gd 155) than in the frontal skin (at gd 172) (Fig 3B and 3F, respectively). In addition, the epidermis of the frontal skin shows signs of immaturity (vacuolization of keratinocytes) up to gd 230 (insert in Fig 4D and 4E), whereas the epidermis of the horn bud is differentiated already at gd 212 (insert in Fig 4A). This might indicate, as suggested in a previous publication [20], that the differentiation of the epidermis and sebaceous glands in the area of the horn bud occurs earlier than the normal skin. However, examination of more samples from the horn bud and normal skin of bovine fetuses at these specific time-points is needed to verify the accuracy of this observation.

Approximately in the third to fourth month of gestation bundles of mesenchymal tissue are visible below the horn bud in the dermis (Fig 2A and 2C). Previously, similar structures were addressed as tissue with glandular/ductal differentiation [16,20]. However, the mesenchymal structures in our samples are positive for NSE, identifying these structures as nervous tissue. Even if nerve fibres of normal size are present in the frontal skin as well as in the area of the horn bud, these thick nerve bundles are not present in the dermis of the frontal skin of wild type fetuses or in the polled fetuses, indicating that the horn bud is a very sensitive area. The development of the bony core of the horn is apparent about 1 month *post partum* [23]. In agreement with this observation, no signs of ossification are present in any of the fetal tissue samples.

## Conclusion

To the best of our knowledge, this is the first study that present unique histological data on bovine prenatal horn bud differentiation at different developmental stages which provides new insights for a better understanding of recent molecular findings.

## Materials and Methods

### Ethics Statement

All animal work was conducted according to the national and international guidelines for animal welfare. The collection of fetal tissue was done at a local governmentally authorized slaughterhouse, as a low number of pregnant cows are routinely slaughtered. The study was approved by the “Cantonal Committee for Animal Experiments” (Canton of Bern; permits BE78/12).

### Animals and sampling

A total of ten fetuses (Table 1) were used for this study. The time of gestation was estimated based on the crown-rump length [24]. In the smallest fetuses (7.7 and 11.3 cm crown rump length, respectively) the entire heads were sampled, fixed in formalin and cut vertically at the site of the horn bud. In the older fetuses (21.5–80 cm crown rump length) a punch biopsy was taken to sample the horn buds and the frontal skin. DNA of the foetuses was isolated from a piece of tail using QIAGEN’s DNeasy kit according to the manufacturers’ instruction. The respective genotypes for the 212 bp insertion-deletion and the Holstein polled mutation was analysed as described [17].

**Table 1:** Fetuses used for histological analysis.

Sample ID	crown-rump length (cm)	sex	estimated age in days	*indel* genotype	genotype Holstein polled mutation
POL1151	7.7	m	70	wild type	wild type
POL1152	11.3	f	83	wild type	wild type
POL1125	21.5	f	115	wild type	wild type
POL1140	29.4	f	140	wild type	wild type
POL1106	34.5	f	155	wild type	wild type
POL1104	42	f	172	wild type	wild type
POL1117	44	f	177	heterozygous	wild type
POL1098	57	m	212	wild type	wild type
POL1122	64	m	230	wild type	wild type
POL1103	80	m	268	wild type	wild type

The fetal age was estimated based on the relation between crown-rump length and time of gestation described by Schnorr and Kressin [24]. They were genotyped for the 212 bp insertion-deletion variant (*indel*) associated with polledness in beef and dual-purpose breeds and for the Holstein polled mutation [17].

### Histological preparation

The tissue samples were fixed in 10% buffered formalin, dehydrated in a graded ethanol series, cleared with xylene and embedded in paraffin. Microtome sections (4 μm) were stained with haematoxylin and eosin (HE). All skin biopsies were histologically evaluated in a blinded fashion. Digital images were obtained with the ProgRes C5 camera.

### Immunohistochemistry

Slides derived from paraffin blocks were stained with NSE (Novocastra, ready to use), CK (MNF116, 1:400, Dako), desmin (1:400, Dako) and SMA (1:5000, Sigma-Aldrich). Immunohistochemistry was performed with an automated stainer and a standardized protocol was used. The specifity of each staining was demonstrated with a positive and a negative control.
